# Supplementary material for: Importance of potassium ions for ribosome structure and function revealed by long-wavelength X-ray diffraction
Source: Nat Commun. 2019 Jun 7;10:2519. doi: 10.1038/s41467-019-10409-4 (PMC6555806; doi:10.1038/s41467-019-10409-4)
Supplement: Supplementary file 1 — Supplementary Information [file 41467_2019_10409_MOESM1_ESM.pdf]

Supplementary Information

Importance of potassium ions for ribosome structure and function revealed by long-wavelength X-ray diffraction

Rozov et al.

Supplementary Figures 1-5

Supplementary Tables 1-4

Supplementary References

SUPPLEMENTARY FIGURES

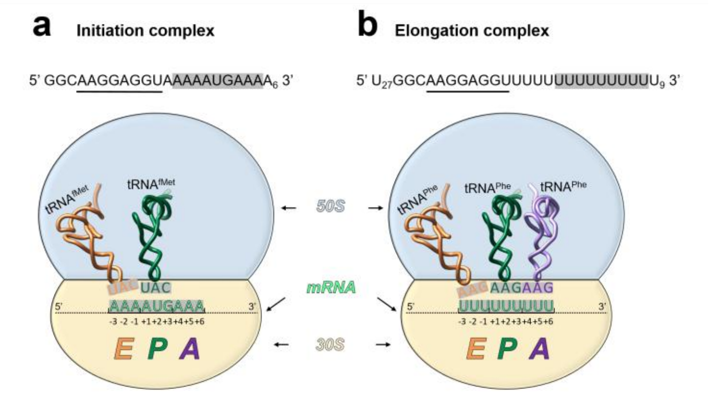

Supplementary Fig. 1 Schematic representation of two 70S complexes used in the study. **a** Initiation complex contained 27 nt mRNA bound with $tRNA_i^{fMet}$ at the P- and E-sites. **b** Elongation complex contained 60 nt poly-Phe mRNA bound with $tRNA^{Phe}$ at the A-, P- and E-sites. The mRNA sequences for both complexes are specified, Shine-Dalgarno sequences are underlined, A-, P-, E-sites triplets are highlighted in green.

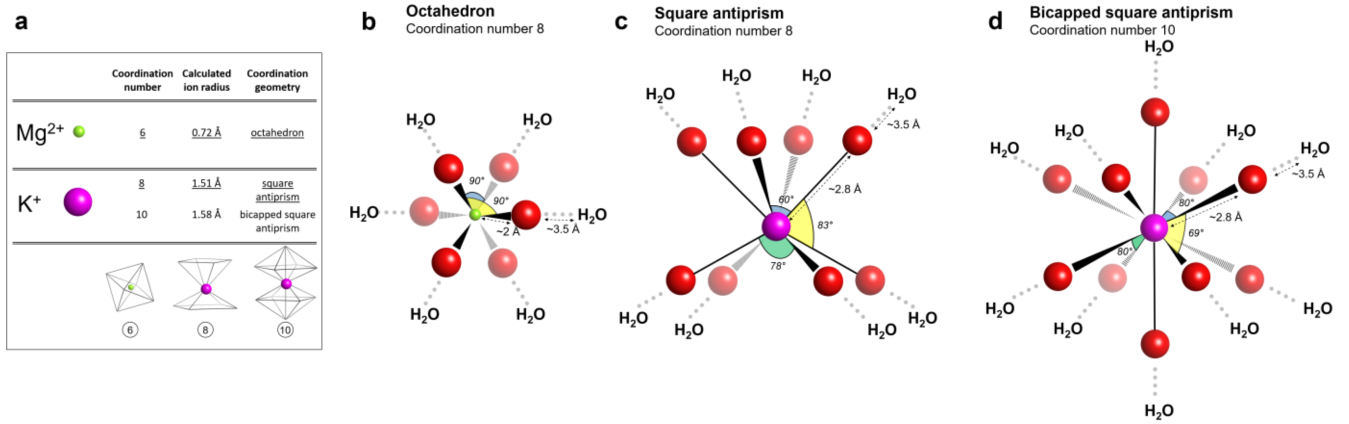

Supplementary Fig. 2 Coordination of Mg^{2+} and K^+ ions most frequently observed in the ribosome. **a** Metal ion coordination and their corresponding calculated radii[1] and geometry. **b-d** Three-dimensional scheme of idealized coordination of Mg^{2+} (green) (**b**) and K^+ (magenta) (**c, d**) with oxygen atoms (red). Potential hydrated shells are shown as grey dots, angles and distances are indicated within the figures.

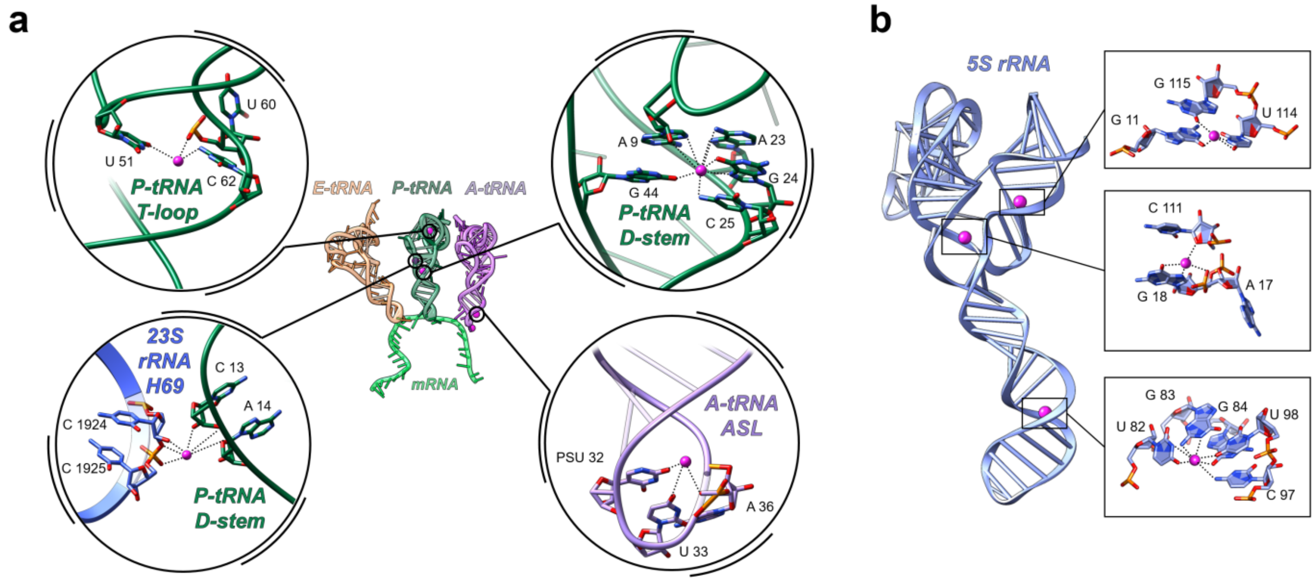

Supplementary Fig. 3. K^+ ions coordinated to tRNAs and 5S rRNA. **a** Binding of tRNAs to the ribosome is stabilized by K^+ in the T-loop, D-stem of the P-site tRNA and anticodon stem loop (ASL) of the A-tRNA. One of the K^+ ions in the D-stem is additionally coordinated with H69 of the 23S rRNA. **b** Three K^+ stabilize 5S rRNA architecture.

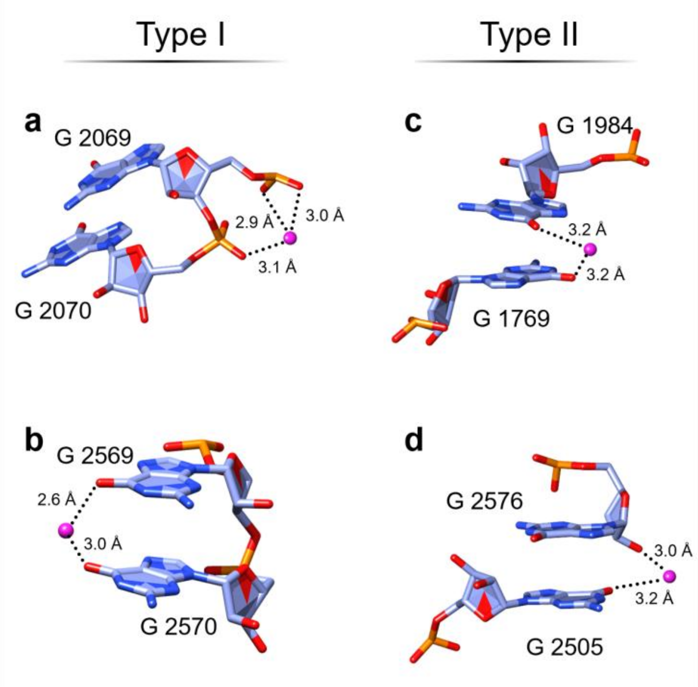

Supplementary Fig. 4 Type I and II stabilization of G-G stacking pair is the most abundant interaction of K^+ ions with the ribosome. Type I represents the class of interaction between two successive guanines in the same strand, either *via* backbone (**a**) or *via* bases (**b**). Type II interaction represents stabilization of distant guanines from different strands, either via bases (**c**), or via base-backbone coordination (**d**).

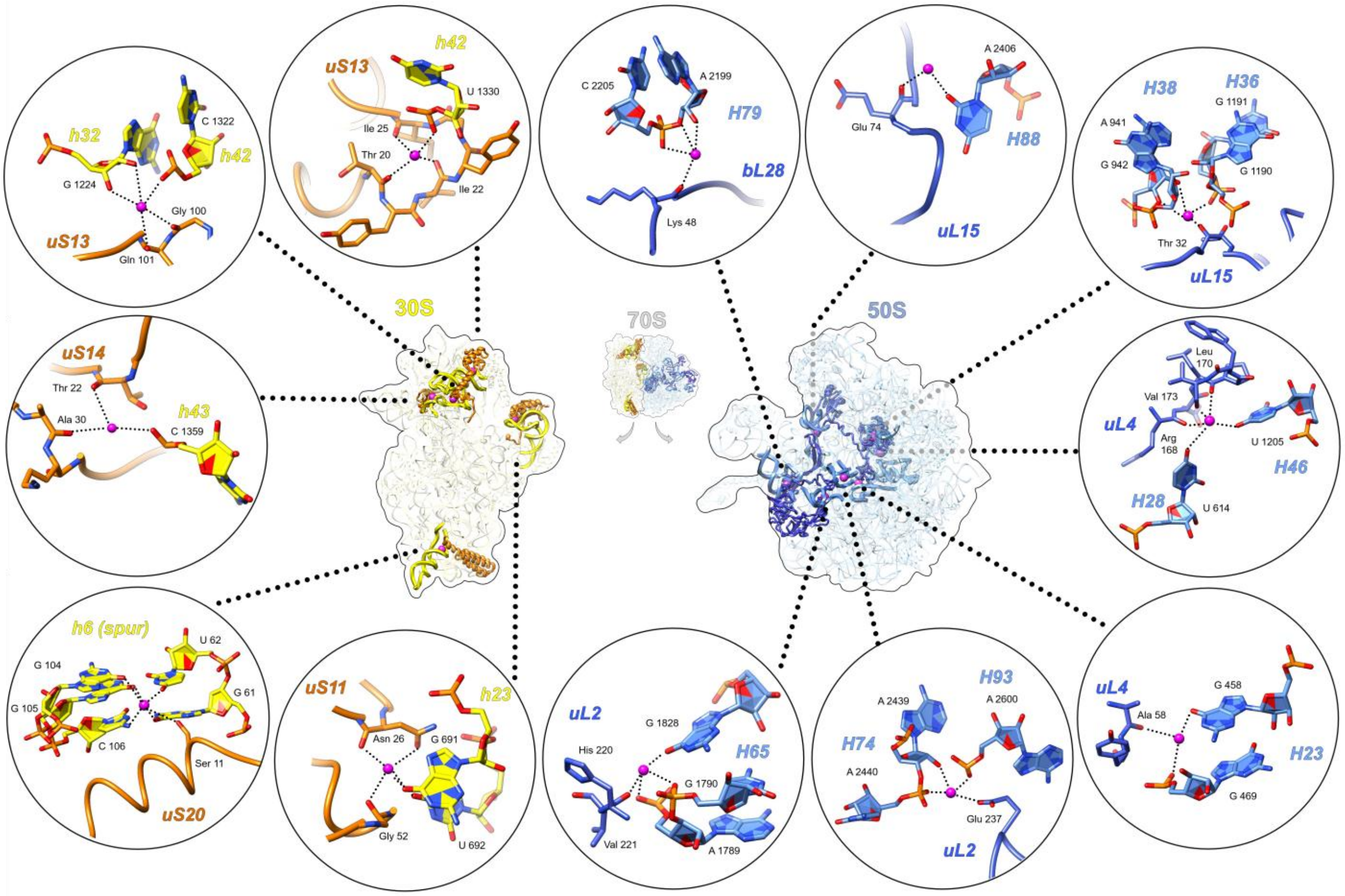

Supplementary Fig. 5 Potassium ions (magenta) that mediate interaction of SSU r-proteins (orange) with 16S rRNA (yellow) and LSU r-proteins (blue) with 23S rRNA (light blue). tRNAs and mRNA are omitted from the figure.

SUPPLEMENTARY TABLES

Supplementary Table 1. Common K^+ ions between analyzed ribosomes. There are two ribosomes within the asymmetric unit arbitrarily named A and B. Along the manuscript, EC vs IC comparison was given for ribosome A of the EC (EC-A) and ribosome B of the IC (IC-B). For determination of overlapping ions, pairwise comparisons between all four ribosomes were performed. Ribosomes were aligned on 23S rRNA. First, proximal K^+ ions with VDW overlap >= -0.5 A were identified using "find clashes/contacts" option in Chimera. Second, all identified proximal K^+ ions were manually inspected and those having the same binding pocket were considered as common ions. The ions with no overlaps were considered as unique.

	IC-A	IC-B	EC-A	EC-B	All common	Unique	Total
**IC-A**		90	139	115	80	22	169
**IC-B**	90		94	98	80	17	127
**EC-A**	139	94		134	80	48	211
**EC-B**	115	98	134		80	8	155

Supplementary Table 2 Data collection and processing statistics for Initiation complex

	Dataset1A	Dataset2A	Dataset1B	Dataset2B
X-ray energy, eV	3700	3700	3500	3500
X-ray wavelength, Å	3.351	3.351	3.542	3.542
Space group	$P2_12_12_1$	$P2_12_12_1$	$P2_12_12_1$	$P2_12_12_1$
Cell dimensions				
a, *b*, *c* (Å)	210.01 449.49 615.49	210.17 450.55 619.07	210.38 448.31 616.21	210.48 450.55 618.84
α, β, γ (°)	90.0 90.0 90.0	90.0 90.0 90.0	90.0 90.0 90.0	90.0 90.0 90.0
Resolution (Å)	362.996-4.65 (4.77-4.65)*	364.287-4.44 (4.56-4.44)	362.52-4.65 (4.77-4.65)	364.24-4.85 (4.98-4.85)
R_{meas}	17.7 (196.9)	20.6 (198.5)	14.4 (198.8)	21.0 (240.2)
$I / \sigma I$	6.85 (0.99)	6.47 (0.98)	8.14 (0.96)	6.11 (0.99)
CC(1/2)**	99.8 (34.1)	99.6 (41.0)	99.9 (42.9)	99.6 (38.0)
Completeness (%)	97.1 (94.6)	98.9 (96.1)	96.5 (92.0)	99.3 (98.0)

*Numbers in parentheses refer to high-resolution shell

**As reported by XSCALE[2,3]

Supplementary Table 3 Data collection and processing statistics for Elongation complex

	Dataset1A	Dataset2A	Dataset3A	Dataset4A
X-ray energy, eV	3700	3700	3700	3700
X-ray wavelength, Å	3.351	3.351	3.351	3.351
Space group	$P2_12_12_1$	$P2_12_12_1$	$P2_12_12_1$	$P2_12_12_1$
Cell dimensions				
a, *b*, *c* (Å)	211.25 448.40 620.12	210.38 448.14 618.18	210.13 450.01 622.23	210.91 447.19 619.62
α, β, γ (°)	90.0 90.0 90.0	90.0 90.0 90.0	90.0 90.0 90.0	90.0 90.0 90.0
Resolution (Å)	363.36-4.41 (4.52-4.41)*	362.83-4.99 (5.12-4.99)	364.641-4.31 (4.42-4.31)	362.615-4.00 (4.10-4.00)
R_{meas}	26.3 (168.3)	28.5 (200.2)	29.1 (158.9)	28.7 (186.5)
$I / \sigma I$	6.28 (1.01)	5.31 (0.99)	5.73 (0.98)	6.08 (1.00)
CC(1/2)**	99.6 (47.6)	99.3 (41.3)	99.4 (32.0)	99.1 (25.3)
Completeness (%)	99.1 (97.6)	99.3 (98.0)	99.2 (97.0)	97.5 (91.4)

*Numbers in parentheses refer to high-resolution shell

**As reported by XSCALE[2,3]

Supplementary Table 3 continued

	Dataset1B	Dataset2B	Dataset3B
X-ray energy, eV	3500	3500	3500
X-ray wavelength, Å	3.542	3.542	3.542
Space group	$P2_12_12_1$	$P2_12_12_1$	$P2_12_12_1$
Cell dimensions			
a, *b*, *c* (Å)	209.41 447.49 619.01	210.40 448.12 618.67	210.15 449.50 621.69
α, β, γ (°)	90.0 90.0 90.0	90.0 90.0 90.0	90.0 90.0 90.0
Resolution (Å)	362.652-5.85 (6.00-5.85)	362.918-4.75 (4.87-4.75)	364.261-5.10 (5.23-5.10)
R_{meas}	27.1 (163.3)	27.2 (156.4)	31.4 (162.5)
$I / \sigma I$	5.37 (1.00)	5.59 (0.99)	4.44 (0.99)
CC(1/2)**	99.8 (40.4)	99.1 (52.6)	99.2 (44.0)
Completeness (%)	99.9 (99.6)	98.6 (97.1)	99.7 (99.1)

*Numbers in parentheses refer to high-resolution shell

**As reported by XSCALE[2,3]

Supplementary Table 4 Model refinement and validation statistics

	Initiation complex	Elongation complex
Dataset PDB ID	4V6G	4V6F
Resolution (Å)	226.09-3.50	223.10-3.10
No. reflections	743746	1041034
Present work PDB ID	6QNQ	6QNR
R_{work} / R_{free}	18.74/25.13	19.64/25.32
No. atoms		
RNA	202857	207167
Protein	94097	94998
Ligand/ion/water	3553	3589
B-factors		
RNA	93.55	73.87
Protein	104.55	80.78
Ligand/ion/water	67.21	48.48
R.m.s. deviations		
Bond lengths (Å)	0.008	0.009
Bond angles (°)	1.403	1.525

SUPPLEMENTARY REFERENCES

1. Lide, D. R. *CRC handbook of chemistry and physics : a ready-reference book of chemical and physical data*. (CRC Press, 2001).
2. Kabsch, W. Xds. *Acta Crystallographica Section D: Biological Crystallography* **66**, 125-132 (2010).
3. Karplus, P. A. & Diederichs, K. Linking crystallographic model and data quality. *Science* **336**, 1030-1033 (2012).
