# Supplementary material for: Importance of potassium ions for ribosome structure and function revealed by long-wavelength X-ray diffraction
Source: Nat Commun. 2019 Jun 7;10:2519. doi: 10.1038/s41467-019-10409-4 (PMC6555806; doi:10.1038/s41467-019-10409-4)
Supplement: Supplementary file 4 — Description of Additional Supplementary Files [file 41467_2019_10409_MOESM4_ESM.pdf]

Description of Additional Supplementary Files

File Name: Supplementary Data 1
Description: **Binding pockets and putative coordination of the K^+ ions discussed in the manuscript.** Selected K^+ ions were analyzed in each ribosome of the two analyzed complexes, IC and EC. Within the asymmetric unit, ribosomes were arbitrarily named A and B. Along the manuscript, EC vs IC comparison was given for ribosome A of the EC (EC-A) and ribosome B from the IC (IC-B). Contacts identified based on van der Waals radii overlap of defined atoms ≥ -0.5 Å. Selection of the coordination is based on the best match between positions of coordinated atoms, estimation of coordination was done using Metal geometry function in UCSF Chimera. Coordination number is shown in square brackets, rmsd indicates the deviation of ligands positions from ideal geometry of selected coordination (n/a – coordination could not be determined). Values are given predominantly for EC-A, or IC-B when EC-A was not applicable. The presence of an ion is marked in grey, the absence in white color.
*Modified RNA nucleotides: OMC – 2'-O-methylcytosine, MIA – 2-methylthio-N6-isopentenyladenosine, 2MA – 2-methyladenosine, 5MC – 5-methylcytosine, 5MU – 5-methyluridine, 7mG – 7-methylguanosine.

File Name: Supplementary Movie 1
Description: Side-by-side comparison of K^+ ions distribution within 70S ribosomes in initiation (IC, left side) and elongation (EC, right side) complexes. The binding of tRNA to the A-site of the ribosome in the EC requires structural mobilization of the decoding center that is assisted by K^+ ions.
